# Recent Advances in the Management of Cancer-Associated Thrombosis: New Hopes but New Challenges

**DOI:** 10.3390/cancers11010071

**Published:** 2019-01-10

**Authors:** Corinne Frere, Ilham Benzidia, Zora Marjanovic, Dominique Farge

**Affiliations:** 1 Institute of Cardiometabolism And Nutrition, INSERM UMRS_1166, Sorbonne Université, F-75013 Paris, France; 2 Department of Haematology, Pitié-Salpêtrière Hospital, Assistance Publique Hôpitaux de Paris, F-75013 Paris, France; 3 Autoimmune and Vascular Disease Unit, Internal Medicine (UF04), Center of reference for rare systemic autoimmne diseases (FAI2R), Saint-Louis Hospital, Assistance Publique Hôpitaux de Paris, F-75010 Paris, France; ilham.benzidia@aphp.fr (I.B.); dominique.farge-bancel@aphp.fr (D.F.); 4 Department of Haematology, Saint-Antoine Hospital, Assistance Publique Hôpitaux de Paris, F-75012 Paris, France; zora.marjanovic@aphp.fr; 5 Department of Medicine, McGill University, Montreal, QC H3A 0E7, Canada

**Keywords:** venous thromboembolism, cancer, low molecular weight heparin, direct oral anticoagulant

## Abstract

Venous thromboembolism (VTE) is a common cause of morbidity and mortality in cancer patients and leads to a significant increase in health care costs. Cancer patients often suffer from multiple co-morbidities and have both a greater risk of VTE recurrence and bleeding compared to non-cancer patients. Anticoagulation is therefore challenging. For many years, long-term therapy with Low-Molecular-Weight Heparin (LMWH) was the standard of care for the management of cancer-associated VTE. Direct oral anticoagulants (DOAC), which offer the convenience of an oral administration and have a rapid onset of action, have recently been proposed as a new option in this setting. Head-to-head comparisons between DOAC and LMWHs for the treatment of established VTE are now available, and data on the efficacy and safety of these drugs for primary prophylaxis of VTE in ambulatory cancer patients receiving systemic anticancer therapy are emerging. This narrative review aims to summarize the main recent advances in the prevention and treatment of cancer-associated VTE, including recent data on the use of individualized factors to stratify the risk of VTE in each individual patient, quality-of-life in patients treated with LMWH, and the place that DOACs will likely take in the cancer-associated VTE management landscape.

## 1. Introduction

Cancer-associated thrombosis (CAT) is a common clinical issue in oncology, and its management often poses a therapeutic challenge for clinicians. Cancer patients treated for established venous thromboembolism (VTE) have a greater risk of VTE recurrence (3-fold) and major bleeding (2-fold) than non-cancer patients [1], with substantial variations in risk across individuals. Several evidence-based clinical practice guidelines (CPGs) have been developed for the management of CAT [2,3,4,5,6,7]. Low-Molecular-Weight Heparin (LMWH) is recommended as the standard of care for the treatment of established VTE in the absence of contraindications [2,3,4,5,6,7], based on five landmark randomized clinical trials (RCTs) demonstrating that LMWH are more effective than vitamin K antagonists (VKA) in this setting, with similar or superior safety profiles [8,9,10,11,12]. A recent Cochrane meta-analysis similarly showed that LMWH reduced the risk of recurrent VTE at 6 months in cancer patients with acute VTE compared to VKA (relative risk (RR) 0.58, 95% confidence interval (CI) 0.43–0.77, risk difference, 53 fewer per 1000), without significant increase in major (RR 1.09; 95% CI, 0.55–2.12) or minor bleeding (RR 0.78, 95% CI 0.47–1.27) [13]. The optimal duration of anticoagulation beyond 6 months remains an unresolved question. Extended duration anticoagulant therapy is usually proposed in cases of active cancer, while taking into consideration individual evaluation of the benefit-risk ratio, tolerability, drug availability, and patient preference. However, despite the high level of evidence supporting LMWH as the first-line anticoagulant in cancer patients, many clinicians have not yet implemented these recommendations into their clinical practice due to several factors, including habit and doubt about patient tolerance, and acceptance of long-term daily subcutaneous injections [14].

Direct oral anticoagulants (DOAC), which include direct thrombin inhibitors (dabigatran) and direct factor Xa inhibitors (rivaroxaban, apixaban, and edoxaban), represent an emerging option in the treatment and prevention of CAT. The pharmacological properties of DOAC circumvent most of the limitations associated with LMWH and VKA. DOAC are administrated orally and are more cost-effective than LMWH. DOAC have a more rapid onset of action and more predictable pharmacodynamics than VKA, precluding the need for dose adjustment and routine monitoring. DOAC are also associated with fewer risks of food-drug and drug-drug interactions compared to VKA. Recently, long-awaited results from dedicated RCTs assessing the safety and efficacy of DOAC in cancer patients have been reported, with several more trials soon to complete. These early trials have reported that DOAC are safe and effective for the treatment and prevention of CAT, bringing a new potential anticoagulant treatment option for cancer patients, but they also raise new treatment challenges in this specific patient population. This narrative review aims to summarize the main recent advances in the prevention and treatment of CAT, including recent data on the use of individualized factors to stratify the risk of VTE in each individual patient, quality-of-life (QoL) in patients treated with LMWH, and the place that DOAC will likely take in the CAT management landscape.

## 2. Treatment of Established CAT: Are DOAC a Safe and Effective Treatment Option?

DOAC are currently endorsed as a first-line treatment for acute deep vein thrombosis (DVT) and pulmonary embolism (PE) in non-cancer patients [7,15,16]. Six large prospective pivotal phase-III RCTs (RE-COVER I and II for dabigatran, EINSTEIN-PE and-DVT for rivaroxaban, AMPLIFY for apixaban, and HOKUSAI for edoxaban) reported that DOAC are non-inferior to VKA, with LMWH bridging therapy for treatment and prevention of recurrent VTE, with similar rates of bleeding [17,18,19,20,21,22]. Each of these phase-III RCTs included a small subset of patients with a history of cancer or an active cancer (3–9%). Post hoc subgroup-analyses performed on these patient subgroups reported similar efficacy and safety results to those obtained in the general population [23,24,25,26,27,28,29]. The DOAC were at least non-inferior to VKA in the prevention of VTE recurrence, with no difference in major bleeding or clinically relevant non-major bleeding (CRNMB). However, these patients may not accurately reflect the true cancer patient population because stringent inclusion criteria for bleeding risk and life expectancy were used, and in some of the trials, cancer patients for whom LMWH treatment was considered appropriate were not eligible for inclusion. Furthermore, only one third of included cancer patients had metastatic disease and less than 40% of them were receiving systemic anticancer therapy. Moreover, the comparator was a VKA, which is not the standard of care in cancer patients. These limitations made these results difficult to interpret and extrapolate to the overall cancer patient population. Accordingly, DOAC are currently not recommended for the treatment of VTE in cancer patients [2,3,5,6,7].

With the recent publication of two dedicated cancer trials, head-to-head comparisons between DOAC and VKA are now available. The Hokusai VTE Cancer trial randomized 1050 cancer patients with acute symptomatic or incidental VTE to receive edoxaban (60 mg daily after at least 5 days of LMWH therapy) or dalteparin (200 IU/kg daily for 1 month, followed by 150 IU/kg daily) for up to 6 to 12 months [30]. Patients having an Eastern Cooperative Oncology Group (ECOG) performance > 2 were excluded from the study. Edoxaban was non-inferior to dalteparin for the composite primary outcome of recurrent VTE or major bleeding during the 12 months after randomization, regardless of treatment duration (Hazard ratio (HR) 0.97, 95% CI 0.7–1.36, *p* = 0.006 for noninferiority, *p* = 0.87 for superiority). The rates of recurrent VTE did not differ between the edoxaban and dalteparin groups (7.9% versus 11.3%, HR 0.71, 95% CI 0.48–1.06, *p* = 0.09), while the rate of major bleeding was significantly higher with edoxaban compared to dalteparin (6.9% vs. 4.0%, respectively, HR 1.77, 95% CI 1.03–3.04, *p* = 0.04). The rates of death did not differ between the two treatment arms (39.5% vs. 36.6% in the edoxaban and dalteparin arms, respectively, HR 1.12, 95% CI 0.92–1.37) [30]. (Table 1) A post-hoc analysis revealed that major bleeding in the edoxaban group predominantly occurred in patients with gastrointestinal cancer (both resected and unresected). In these patients, the risk of major bleeding was significantly higher in patients treated with edoxaban compared to those treated with dalteparin (12.5% vs. 3.6%, HR 4.0, 95% CI 1.5–10.6, *p* = 0.005) [31].

The recently published SELECT-D pilot trial randomized 406 cancer patients with symptomatic or incidental VTE having an ECOG performance status ≤2 to receive rivaroxaban (15 mg twice daily for 3 weeks followed by 20 mg once daily) or dalteparin (200 IU/kg daily for 1 month followed by 150 IU/kg daily) for 6 months. At 6-month follow-up, the cumulative rate of recurrent VTE was significantly lower with rivaroxaban compared to dalteparin (4% vs. 11%, HR 0.43, 95% CI 0.19–0.99). The cumulative rate of major bleeding did not differ between the two treatment groups (6% vs. 4% in the rivaroxaban and dalteparin arms respectively, HR 1.83, 95% CI 0.68–4.96), while the cumulative rate of CRNMB was significantly higher in patients treated with rivaroxaban compared to dalteparin (13% vs. 4%, respectively, HR 3.76, 95% CI 1.63–8.69). Overall 6-month survival did not differ between the two treatment arms (75% vs. 70% in the rivaroxaban and dalteparin arms, respectively) [32]. In patients with esophageal or gastroesophageal cancer, major bleeding tended to occur more frequently with rivaroxaban than with dalteparin (36% vs. 11%, in the rivaroxaban and dalteparin arms, respectively) (Table 1) [32]. Most major bleeding events in the rivaroxaban group (7 out of 11) were gastrointestinal. Most CRNMB events occurring in patients treated with rivaroxaban were gastrointestinal (9 out of 25) or genitourinary (11 out of 25).

The rates of recurrent VTE observed in the LMWH arm of both the Hokusai VTE Cancer trial (11.3%) and the SELECT-D pilot trial (11%) were consistent with those observed in the landmark studies comparing long-term treatment with LMWH to VKA in cancer patients (CLOT (9%), LITE (7%), CATCH (7.2%)). The rates of major bleeding in both trials (4%) were also comparable to those noted in CLOT (6%), LITE (7%), and CATCH (3%) [9,11,12]. The cancer patients included in the Hokusai VTE Cancer and SELECT-D trials reflected “real world” oncology practice, with more than half of participants having a metastatic disease (53% and 58%, respectively), of which almost two-thirds were receiving systemic anticancer therapy (72 and 70%, respectively).

A recent meta-analysis pooling results from both the Hokusai VTE Cancer and SELECT-D trials reported a numerically lower rate of VTE recurrence in patients treated with DOAC compared to dalteparin, but this did not reach statistical significance (RR 0.65, 95% CI 0.42–1.01, I² 17%). However, the rates of major bleeding (RR 1.74, 95% CI 1.05–2.88, I² 0%) and CRNMB (RR 2.31, 95% CI 0.85–6.28, I² 78%) were significantly higher in patients treated with DOAC compared to those treated with dalteparin, indicating a need for caution [33].

Based on these findings, the Scientific and Standardization Committee on Hemostasis and Malignancy of the International Society on Thrombosis and Hemostasis (ISTH) recently released guidance on the current role of DOAC in the treatment of CAT [34]. Edoxaban and rivaroxaban are suggested for cancer patients with established VTE, who are at low risk of bleeding, and who have no potential drug-drug interactions with concurrent systemic anticancer therapy. The guidance emphasizes the importance of physician-patient shared-decision making, which takes into account patient preferences and values.

In December 2018, the results of the Phase-IV ADAM-VTE trial (NCT02585713) were presented at the 60th American Society of Hematology annual meeting [35,36]. Three hundred cancer patients having an ECOG performance status ≤2 with acute symptomatic or incidental VTE were randomized to receive apixaban (10 mg twice daily for 7 days followed by 5 mg twice daily) or dalteparin (200 IU/kg daily for 1 month followed by 150 IU/kg daily) for 6 months. The primary safety endpoint, rate of major bleeding, was similar in the two treatment groups (0.0% (0 out of 145 patients) in the apixaban arm vs. 2.1% (3 out of 142 patients) in the dalteparin arm, *p* = 0.9956). The rates of the secondary safety composite endpoint (major and CRNM bleeding) were also similar for both groups (9%). VTE recurrence rate was significantly lower with apixaban compared to dalteparin (3.4% (5 out of 145 patients) vs. 14.1% (20 out of 142 patients) in the apixaban and dalteparin arms, respectively, HR 0.26, 95% CI 0.09–0.80, *p* = 0.0182). The rates of death did not differ between the two treatment arms (15.9% vs. 10.6% in the apixaban and dalteparin arms, respectively, HR 1.36, 95% CI 0.79–2.35) (Table 1). QoL surveys in the ADAM-VTE trial revealed a better tolerance to apixaban compared with dalteparin [35,36]. Among cancer patients included in ADAM-VTE trial, 65.5% had a metastatic disease and 74% received systemic anticancer therapy. The study was powered to detect a decrease in 6-month major bleeding rate of 6% with dalteparin (as observed in the LMWH arm of landmark studies) to 1.4% with apixaban. However, no major bleeding occurred in patients treated with apixaban, and the rate of major bleeding in the dalteparin group was lower than predicted (2.1%). The difference in risk of recurrent VTE between DOAC and LMWH treatment in the ADAM-VTE trial (−10.7%) was higher than those observed in the Hokusai VTE Cancer (−3.4%) and SELECT-D trials (−7.0%), whereas the rate of recurrent VTE in the LMWH arm of ADAM-VTE (14.1%) was higher than those observed in Hokusai VTE Cancer (11.3%) and SELECT-D (11%). Despite the small sample size and the limitations of the ADAM-VTE trial, these results suggest a favorable risk–benefit ratio for apixaban in the treatment of acute VTE in the cancer patients.

Results of additional ongoing head-to-head trials comparing DOAC to LMWH for the long-term treatment of acute VTE in cancer patients are awaited to shed more light on the efficacy and safety of DOAC in this setting. The CARAVAGGIO trial (NCT03045406) will randomize 1168 cancer patients with acute VTE to receive apixaban (10 mg twice daily for 7 days followed by 5 mg twice daily) or dalteparin (200 IU/kg daily for 1 month followed by 150 IU/kg daily) for 6 months [37]. The primary efficacy endpoint is the rate of objectively confirmed recurrent VTE and the primary safety outcome is the rate of major bleeding. This study, for which recruitment is almost completed, will be the largest trial comparing a DOAC to LMWH in cancer patients with acute VTE.

## 3. Primary Prophylaxis of CAT in Ambulatory Patients Receiving Chemotherapy: Which Steps Forward?

Up to 74% of all CAT events occur in the outpatient setting [38]. A retrospective analysis of patients from the United States (USA) IMPACT health care claims database reported that the cumulative incidence of VTE 3.5 months after starting chemotherapy was 7.3% (range 4.6–11.6%), and 13.5% by 12 months (range 9.8–21.3%), and that these rates varied depending on cancer location [39].

Two large RCTs found that primary thromboprophylaxis with LMWH in ambulatory patients receiving chemotherapy significantly decrease the risk of symptomatic VTE compared to placebo, without increasing the risk of major or CRNMB [40,41]. A recent update of a Cochrane review reported that primary thromboprophylaxis with LMWH significantly reduced the rate of symptomatic VTE in ambulatory cancer patients receiving chemotherapy compared to no prophylaxis (RR 0.54, 95% CI 0.38–0.75) without significantly increasing the risk of major bleeding (RR 1.44, 95% CI 0.98–2.11) [42]. This review demonstrated that, assuming a risk of 7.1 symptomatic VTE events per 100 patients, 30 (95% CI 23–56) patients would need to be treated to prevent a single event, suggesting that primary prophylaxis should not be routinely used in cancer patients. Risk assessment models (RAM), designed to identify patients at high risk of VTE, may help to guide decision making in this setting [42]. RCTs in specific cancers associated with a high VTE risk have reported more favorable risk-benefit ratios. LMWH dramatically reduced the rate of VTE occurrence in patients with pancreatic cancer in the FRAGEM study (RR 0.42, 95% CI 0.19–0.94, *p* = 0.039) [43] and in the PROSPECT-CONKO 004 study (HR 0.40, 95% CI 0.19–0.83, *p* = 0.01) [44]. Based on this evidence, the international initiative on thrombosis and cancer (ITAC) CPGs recommend that primary prophylaxis should not be used routinely, but that it may be indicated in patients with locally advanced or metastatic pancreatic or lung cancer treated with chemotherapy who are at a low risk of bleeding [6].

Owing to the importance of VTE risk in the decision to provide primary prophylaxis, attempts have been made to improve patient risk stratification. The Khorana score, which was based on a collection of readily available clinical (type of cancer, body mass index ≥ 35 k/m²) and biological parameters (platelet count > 350,000/µL, leucocyte count > 11,000/µL, hemoglobin < 10 g/dL or use of erythropoiesis-stimulating agent) was first developed to assess VTE risk in patients receiving chemotherapy [45]. The Khorana RAM was later validated in independent studies [46]. The Khorana score identifies low VTE-risk patients (score of 0), intermediate VTE-risk patients (score of 1–2), and high VTE-risk patients (score ≥ 3). Several modifications of the Khorana score have been developed to further improve its predictive power [47,48,49,50]. Other risk assessment models have recently been proposed, including the COMPASS-CAT score, for use in breast, colorectal, lung, and ovarian cancer [51], and the TiC-Onco score, which combines genetic and clinical risk factors [52]. Recently, Pabinger et al. developed and externally validate simple RAM that included only 2 variables: (1) tumor site category (very-high and high vs. intermediate or low) and (2) D-dimer levels as continuous variables [53]. This model demonstrated better predictive strength, but these findings require further confirmation [53].

Recently, the multicenter randomized PHACS study provided a proof of concept of the benefit of primary thromboprophylaxis in high VTE-risk ambulatory cancer patients receiving chemotherapy. Cancer patients initiating a new systemic anticancer regimen with a Khorana score ≥ 3 were screened for VTE at baseline and included if negative. One hundred and seventeen patients were randomized to either receive dalteparin 5000 IU daily or no treatment for 3 months. Subjects were screened with lower extremity ultrasounds every 4 weeks while on the study and with chest computed tomography at 12 weeks. VTE occurred in 12% of patients receiving dalteparin vs. 21% in patients who went untreated (HR 0.69, 95% CI 0.23–1.89). The corresponding number needed to treat to prevent a VTE event was 12 [54]. However, these results should be interpreted with caution, since the use of screen-detected VTE as an endpoint represents a major limitation of the study.

DOAC are not licensed for primary prophylaxis of VTE, except after elective major orthopedic surgery. Data on their efficacy and safety for primary prevention of VTE in cancer patients have been limited, but new data have recently become available. The Phase-II pilot ADVOCATE study randomized 125 cancer patients treated with systemic anticancer therapy to either receive a once-daily doses of apixaban (5 mg, 10 mg, or 20 mg), or placebo. No subject receiving apixaban developed symptomatic VTE in any of the 3 dosage groups (5 mg, 10 mg, or 20 mg), compared to 3 out of 29 patients (10.3%) in the placebo group. No major bleeding occurred in the 5 and 10-mg apixaban groups, 2 major bleeding events occurred in the 20-mg apixaban group, and 1 occurred in the placebo group [55]. Two recent dedicated RCTs have assessed the safety and efficacy of DOAC for primary VTE prevention in ambulatory cancer patients receiving systemic anticancer therapy. Both studies focused on patients identified as being at intermediate or high-risk of VTE, with the well-validated Khorana score. The CASSINI trial (NCT02555878) randomized 841 cancer patients (Khorana score ≥ 2), initiating chemotherapy to either receive rivaroxaban (10 mg once daily) or placebo for 6 months [56]. Patients with primary or metastatic brain cancer, and those at high risk of bleeding, were excluded. Over the entire 6-month follow-up, the composite primary endpoint of DVT, PE, and VTE-related death occurred in 5.95% of patients in the rivaroxaban group and 8.79% in the placebo group (HR 0.66, 95% CI 0.40–1.09, *p* = 0.101, NNT = 35). However, during the on-treatment period, patients on rivaroxaban experienced fewer primary endpoint events compared to patients on placebo (HR 0.40, 95% CI 0.20–0.80, *p* = 0.007; NNT = 26). The rate of major bleeding and CRNMB did not differ between the two treatment arms (HR 1.96, 95% CI 0.59–6.49, *p* = 0.265 and HR 1.34, 95% CI 0.54–3.32, *p* = 0.53). All-cause mortality rates were similar between groups (20.0% in patients on rivaroxaban vs. 23.8% in patients on placebo, HR 0.83, 95% CI 0.62–1.11, *p* = 0.213). (Table 2)

The AVERT trial (NCT02048865) randomized 574 cancer patients at intermediate or high-risk of VTE (Khorana score, ≥2) who were initiating chemotherapy to receive apixaban (2.5 mg twice daily) or placebo for 6 months [57]. Patients with acute leukemia or myeloproliferative neoplasms, and those at increased risk of clinically significant bleeding were excluded. At 6-month follow-up, the rate of objectively-confirmed VTE, the primary efficacy outcome, was significantly lower in the apixaban arm compared to placebo (4.2% vs. 10.2%, HR 0.41, 95% CI 0.26–0.65, *p* < 0.001). The rate of major bleeding, the primary safety outcome, was significantly higher in the apixaban group compared to placebo (3.5% vs. 1.8%, HR 2.00, 95% CI 1.01–3.95, *p* = 0.046). The rate of CRNMB, the secondary safety outcome, did not differ between the two treatment arms (7.3% in the apixaban arm vs. 5.5% in the placebo arm, HR 1.28, 95% CI 0.89–1.84). Rates of death from any cause were similar between the two treatment arms (12.2% in the apixaban arm vs. 9.8% in the placebo arm, HR 1.29, 95% CI 0.98–1.71) (Table 2).

## 4. Unsolved Issues and Challenges

### 4.1. Duration of Anticoagulation

Determining the optimal duration of anticoagulation in the treatment of established CAT remains an unresolved issue. RCTs that compared LMWH to VKA for the treatment of acute VTE in this setting followed the participants for only 3–6 months. CANTHANOX was the first study to demonstrate superiority of LMWH over VKA in 146 cancer patients with acute VTE, based on a composite primary outcome measure of major bleeding or recurrent VTE at 3 months [8]. Two larger RCTs (CLOT [9] and CATCH [12]) compared LMWH to VKA for a duration of 6 months. In the CLOT trial, the rate of recurrent VTE at 6 months was significantly lower in the dalteparin arm compared to the warfarin arm (9% vs. 17% in the dalteparin and warfarin arms, respectively; HR, 0.48; 95% CI, 0.30–0.77; *p* = 0.002). The rate of major bleeding was similar between the two arms (6% vs. 4% in the dalteparin and warfarin arms, respectively; *p* = 0.27). In the CATCH trial [12], the rate of recurrent VTE at 6 months was numerically lower in the tinzaparin group (6.9%) compared to warfarin (10.5%), but this difference did not reach statistical significance (HR, 0.65; 95% CI, 0.41–1.03 *p* = 0.07). The rate of major bleeding did not differ between the two treatment groups (2.7% vs. 2.4% in the tinzaparin and warfarin arms, respectively; HR 0.89, 95% CI 0.40–1.99, *p* = 0.77). Based on available evidence, the current international ITAC guidelines recommend that LMWH be used for a minimum of 3 months to treat established VTE in patients with cancer. Although the two largest studies in this setting treated patients for 6 months [9,12], the strength of the evidence supporting treatment up to 6 months is low.

An extended duration of anticoagulant therapy is usually proposed for patients with active cancer, since the risk of recurrent VTE remains high as long as the cancer is active [2,3,5,6,7]. A retrospective cohort study of 358 cancer patients treated for acute VTE showed that in patients in whom anticoagulant treatment was stopped for reasons other than major bleeding, the rate of recurrent VTE was higher in those with an active cancer (19-per-100 patient-years, median duration of 5.8 months) than those with cured cancer (3.2-per-100 patient-years, median duration of 6.0 months) [58]. These results demonstrated that in cases of active cancer, a high risk of recurrent VTE persists beyond the initial 6 months of anticoagulant therapy. Several studies have identified individual risk factors for recurrent VTE or bleeding complications, including age <65 years, female gender, prior history of VTE, diagnosis of malignancy within 3 months of VTE onset, locally advanced or metastatic disease, and lung or hepatobiliary cancers, were all associated with VTE recurrence in cancer patients [1,59,60,61,62,63]. The Ottawa score, which is based on readily accessible clinical variables, was developed as a risk stratification model for predicting risk of VTE recurrence in cancer patients under anticoagulant therapy [60], but validation studies found conflicting data regarding its predictive power [64,65,66]. Retrospective analyses of the RIETE registry reported that the clinical course of VTE-related outcomes may differ according to the type of cancer, underscoring the potential interest for cancer-specific anticoagulant strategies in breast, prostate, colorectal, lung, and pancreatic cancer patients [67,68].

Only two prospective multicenter studies have assessed the safety and efficacy of extended therapy with LMWH up to 12 months in cancer patients with acute VTE. Both studies have reported a good safety profile of extended therapy [69,70]. In the DALTECAN study, 109 out of 334 patients completed 12 months of anticoagulation with dalteparin (200 IU/kg daily followed by 150 IU/kg daily after thereafter). The rate of major bleeding was 3.6% per patient-month in the first month, 1.1% during months 2–6, and 0.7% during months 7–12. The incidence rate of recurrent VTE was 5.7% in the first month, 3.4% during months 2–6, and 4.1% during months 7–12 [69]. The TiCAT study enrolled 247 cancer patients with acute VTE treated with tinzaparin (175 IU/Kg) up to 12 months. The rate of clinically relevant bleeding was 0.9% per patient-month during months 1–6 and 0.6% per patient-month during months 7–12 (*p* = 0.5). The rate of VTE recurrence was 4.5% during months 1–6 and 1.1% during months 7–12 [70]. Overall, these results suggest a favorable benefit-risk ratio for extended duration of treatment.

A recent propensity score-matched cohort study compared the safety and efficacy outcomes in 482 cancer patients receiving LMWH beyond the first 6 months and in 482 cancer patients switched to VKA after the first 6 months [71]. During approximately 9 months of anticoagulant therapy (mean 275.5 days), the rate of recurrent DVT (relative risk (RR) 1.41; 95% CI, 0.68–2.93), recurrent PE (RR 0.73; 95% CI, 0.34–1.58), major bleeding (RR 0.96; 95% CI, 0.51–1.79), and nonmajor bleeding (RR 1.15; 95% CI, 0.55–2.40) were similar in patients who continued LMWH compared with those who switched to VKA [71].

The ongoing APIxaban Cancer Associated Thrombosis (API-CAT, NCT03692065) study aims to assess the efficacy and safety of extended anticoagulant treatment with 2 apixaban doses (5 mg twice daily or 2.5 mg twice daily) in patients with active cancer and VTE who have completed 6 months of anticoagulant therapy with LMWH.

### 4.2. The Choice Paradox: More Options Also Mean Increased Complexity

Given the emergence of high quality evidence supporting the use of DOAC as an option for the prevention and treatment of CAT, clinicians are now facing a multitude of choices for each individual patient. Choosing an anticoagulant and the appropriate dose in cancer patients who often suffer from multiple co-morbidities has become increasingly complex, resulting in uncertainty about whether specific agents should be preferred over others.

LMWHs are administrated subcutaneously, while DOAC offer the convenience of an oral administration. Both LMWH and DOAC are available in different dosages and daily dosing regimens. Strategies within the first days of anticoagulation also differ both among LMWH and among DOAC. For instance, dalteparin is administered at dose of 200 IU/kg daily during the first month and at dose of 150 IU/kg daily thereafter. An initial 5-days course of parenteral therapy is required before initiating dabigatran or edoxaban, while apixaban and rivaroxaban are given with no need for initial course of parenteral therapy but at higher dose during the first 21 days for rivaroxaban (15 mg twice daily), and during the first 7 days of treatment for apixaban (10 mg twice daily). Considering pharmacokinetic characteristics of anticoagulants is warranted when choosing one agent over another (Table 3). Moreover, cost of drugs, regulatory approval, and affordability may influence the choice.

In cases of renal impairment, specific dose adjustment criteria are recommended for each DOAC according to the level of renal impairment, but they differ across DOAC, which may result in prescription errors. Both LMWHs and DOAC are contraindicated in patients with a CrCl <30 mL/min, in which case only unfractionated heparin (UFH) should be used. Of note, UFH is very rarely used in patients with severe renal impairment in daily practice and most patients are being treated with LMWH, with the dose being adjusted based on anti-Factor Xa-levels.

Finally, a significant concern when choosing an anticoagulant is potential drug-drug interactions. Numerous systemic anticancer drugs that are potent inhibitors or inducers of P-glycoprotein and Cytochrome P450 CYP3A4 or CYP2J2 or breast cancer resistance protein may interfere with DOAC, while no drug-drug interactions are expected with LMWH [72,73,74]. For example, antimitotic microtubule inhibitors (e.g., vinca alkaloids and taxanes), tyrosine kinase inhibitors (Imatinib, dasatinib, nilotinib, lapatinib, sunitinib, crizotinib, vemurafenib, vandetanib), some immune-modulating drugs (cyclosporine, dexamethasone, tacrolimus), aprepitant, and fosaprepitant, which are used in numerous emetogenic chemotherapy regimens, interact with CYP3A4, P-glycoprotein, or both [72].

### 4.3. Ensuring Adherence and Education

In daily practice, adherence to CPG guidelines remains low worldwide [75]. Real-world data indicate that oral anticoagulation with either VKA or DOAC is frequently used. A recent retrospective analysis of 6345 cancer patients included in the European Registro Informatizado de Enfermedad TromboEmbólica (RIETE) registry found that only 66% of patients had received long-term LMWH therapy for acute VTE, while 24% of them had been switched to VKA within the first week, and 9.8% beyond the first week [76]. Analyses of US healthcare insurance claims have recently highlighted an increase in the use of DOAC. Among 2941 cancer patients with VTE from the Humana Database medical and pharmacy claims, only 25% of patients received long-term LMWH therapy, while 47.7% were switched to VKA and 24.1% received rivaroxaban [77]. Further analyses of the same database found a shorter treatment duration with LMWH (1 month) compared to VKA (3.5 months) and DOAC (3 months), suggesting a better adherence with oral anticoagulants [78,79]. The recent CAT AXIS multicenter cross-sectional study investigated adherence to thromboprophylaxis in outpatients receiving antineoplastic treatment based on a case vignette methodology. The overall rate of thromboprophylaxis was 32.6% and ECOG index, metastatic disease, on-going chemotherapy, and history of thrombosis were the main factors influencing the decision to use thromboprophylaxis [80].

There are three main reasons for suboptimal adherence to LMWH: (1) fear of potential side effects, including heparin-induced thrombocytopenia (HIT), allergic reactions, pain, and ecchymosis at injection sites, (2) preconceptions about the overall impact of LMWH on QoL, and (3) drug costs. The incidence of thrombocytopenia in cancer patients receiving long-term LMWH or VKA was similar in the landmark studies that compared LMWH to VKA for at least 3 months and up to 6 months [8,9,10,11,12]. Risk of allergic reaction was also similar between the LMWH and placebo arms in the MALT study [81]. Therefore, only pain and development of indurations at injection sites, which can develop in 30–90% of patients [82], can be critical points regarding patient compliance and adherence to treatment. Few studies have assessed QoL in cancer patients with VTE treated with anticoagulant treatment. When cancer patients were specifically questioned on anticoagulant decision making in this setting, avoiding interference with their cancer treatment was the most important factor, followed by safety and efficacy with minimizing the bleeding risk, and were in all cases of greater importance over oral or injected route of administration [83]. In the TROPIQUE study [84], among 409 cancer patients with VTE, mean LMWH treatment duration was 6.27 ± 0.15 months and 98.0% of patients were treated for at least 3 months or more. Patients’ expectations were blood clot prevention, symptom relief, and ease of use. LMWH treatment appeared convenient and 69.1% of patients were satisfied or very satisfied, particularly on measures of reassurance about treatment efficacy and experience with side effects. QUAVITEC, a second prospective, longitudinal, multicenter study recruited 400 consecutive eligible adult cancer patients with VTE who agreed to answer 3 QoL questionnaires (the Medical Outcome Study 36-item Short-Form Health Survey (MOS SF-36) for generic Health- Related Quality of Life (HRQoL), the European Organization for Research and Treatment of Cancer Quality of Life Questionnaire (EORTC QLQ-C30), and the Venous Insufficiency Epidemiological and Economic Study (VEINES-QOL) questionnaire, at anticoagulant treatment initiation (M0), then at 3 (M3) and 6 (M6)-months of follow-up [85]. During follow-up, 18.9% of patients on LMWH reported at least one side effect of injections, consisting of either pain at injection site (7.3%), ecchymosis (16.1%), pruritis (0.6%), or nodules (7.9%). QoL scores in the MOS SF-36 (*p* < 0.0001) and EORTC QLQ-C30 (*p* < 0.0001) questionnaires significantly improved over the 6-month study period in patients treated with LMWH, while VEINES-QOL scores did not change. Factors pertaining to reduced mobility were also identified as significant predictors of QoL outcomes, including being bedridden in the MOS SF- 36 and ECOG score ≥ 2 in the EORTC QLQ-C30. The QUAVITEC study showed that LMWH did not hinder QoL improvements in cancer patients who survived to 6-month follow-up and who exhibited increased health overall. Given how crucial anticoagulant treatment is to decreasing morbidity and mortality, these data contribute to dispelling concerns about the negative impact of LMWH treatment regimens on overall patient well-being and QoL [85]. Furthermore, it has been demonstrated that LMWH is similar to oral anticoagulant regarding cost per quality adjusted life year [86].

Of note, a strict adherence is required to obtain a stable anticoagulation with DOAC, given their short half-lives. Therefore, patient education is crucial [87].

## 5. Conclusions

With the emergence of DOAC as a new option for the prevention and treatment of CAT, clinicians are now facing a multitude of choices for each individual patient. A personalized approach, matching the right drug to the right patient based on drug properties, efficacy, and safety, the side effect profile of each drug, and patient preference will thus probably supplant the one size fit all approach in the future.

## Figures and Tables

**Table 1:** Direct oral anticoagulant (DOAC) vs. Low-Molecular-Weight Heparin (LMWH) for Long-term Treatment of VTE in Patients with Cancer: Data from Prospective Randomized Trials.

Study	Hokusai Cancer VTE		SELECT-D		ADAM-VTE	
**VTE index**	symptomatic or incidental VTE		symptomatic or incidental VTE		symptomatic or incidental VTE	
**Study Period**	12 months		6 months		6 months	
**Treatment arm**	edoxaban	dalteparin	rivaroxaban	dalteparin	apixaban	dalteparin
**Patients characteristic**						
**Age, years**	Mean (SD) = 64.3 (11.0)	Mean (SD) = 63.7 (11.7)	Median (Range) = 67 (22–87)	Median (Range) = 67 (34–87)	-	-
**Metastatic disease (%)**	52.5	53.4	58	58	-	-
**Anticancer therapy (%)**	71.6	73.1	69	70	-	-
**Recurrent VTE (%)**	7.9	11.3	4	11	3.4	14.1
	HR 0.71, 95% CI 0.48–1.06 *p* = 0.09		HR 0.43, 95% CI 0.19–0.99 *p* = NR		HR 0.26, 95% CI 0.09–0.80 *p* = 0.0182	
**Major bleeding (%)**	6.9	4	6	4	0	2.1
	HR 1.77, 95% CI 1.03–3.04 *p* = 0.04		HR 1.83, 95% CI 0.68–4.96 *p* = NR		*p* = 0.9956	
**CRNMB (%)**	14.6	11.1	13	4	6.2	4.2
	HR1.38, 95% CI 0.98–1.94 *p* = NR		HR 3.76, 95% CI 1.63–8.69 *p* = NR		NR	
**Mortality (%)**	39.5	36.6	25	30		
	HR 1.12; 95% CI 0.92–1.37		NR		HR 1.36, 95% CI 0.79–2.35	

CI, confidence interval; CRNMB, clinically relevant non-major bleeding; DVT, deep vein thrombosis; HR, hazard ratio; NR, not reported; PE, pulmonary embolism; SD, standard deviation; VTE, venous thromboembolism.

**Table 2:** DOAC vs. placebo for primary prophylaxis of VTE in Patients with cancer. Data from Prospective Randomized Trials.

Study	CASSINI		AVERT	
Patients	Cancer patients with a Khorana score ≥2 who were initiating chemotherapy Patients with primary or metastatic brain cancer and those at risk for bleeding were excluded		Cancer patients with a Khorana score ≥2 who were initiating chemotherapy Patients with basal cell carcinoma, squamous cell carcinoma, acute leukemia, or myeloproliferative neoplasms and those at increased risk of clinically significant bleeding were excluded	
Study Period	6 months		6 months	
Treatment arm	rivaroxaban	placebo	apixaban	placebo
VTE (%)	2.62 *	6.41 *	4.2	10.2
	HR 0.40, 95% CI 0.20–0.80 *p* = 0.007		HR 0.41, 95% CI 0.26–0.65 *p* < 0.001	
Major bleeding (%)	1.98	0.99	3.5	1.8
	HR 1.96, 95% CI 0.59–6.49 *p* = 0.265		HR 2.00, 95% CI 1.01–3.95 *p* = 0.046	
CRNM bleeding (%)	2.72	1.98	7.3	5.5
	HR 1.34, 95% CI 0.54–3.32 *p* = 0.53		HR 1.28, 95% CI 0.89–1.84 *p* = NR	
Mortality (%)	20.0	23.8	12.2	9.8
	HR 0.83, 95% CI 0.62–1.11 *p* = 0.213		HR 1.29, 95% CI 0.98–1.71 *p* = NR	

* VTE and VTE related death while on-treatment. CI, confidence interval; CNRM, clinically relevant non-major; HR, hazard ratio; NR, not reported; VTE, venous thromboembolism.

**Table 3:** Comparison of the Pharmacologic characteristics of Low-molecular-weight heparin with those of available direct oral anticoagulants.

Characteristics	LMWH	Dabigatran	Rivaroxaban	Apixaban	Edoxaban
**Prodrug**	No	Yes	No	No	No
**Bioavailability (%)**	90	3–7 (pH dependent-Tartaric acid added into the dabigatran capsule)	≥80 when taken with food (for 15 and 20 mg dosing)	50 (Food independent)	62 (Food independent)
**Tmax (h)**	3–4	1–3	2–4	3–4	1–2
**Half-life (h)**	4–6	12–17	5–13 (age dependent)	9–14	10–14
**Excretion**	Renal excretion	Urine (80%)	Urine (66% (~36% as unchanged drug; 30% as inactive metabolites)); feces (28% (7% as unchanged drug; 21% as inactive metabolites))	Urine (~27% as parent drug); feces (biliary and direct intestinal excretion)	Urine (primarily unchanged); renal clearance: ~50% of total clearance
**Metabolism**	Partially metabolized by desulphatation and depolymerization	Hepatic; dabigatran etexilate rapidly and completely hydrolyzed to dabigatran (active form) by plasma and hepatic esterases; dabigatran undergoes hepatic glucuronidation to active acylglucuronide isomers	Hepatic via CYP3A4/5 and CYP2J2	Hepatic predominantly via CYP3A4/5 and to a lesser extent via CYP1A2, 2C8, 2C9, 2C19, and 2J2 to inactive metabolites; -demethylation and hydroxylation are the major sites of transformation; substrate of P-gp and BCRP	Minimal via hydrolysis, conjugation and oxidation by CYP3A4; predominant metabolite (M-4) is active (<10% of parent compound)
**Transporter involved**	-	P-gp (dabigatran etexilate only)	P-gp, BCRP	P-gp, BCRP	P-gp
**Specific antidot**	Protamine (partial)	Idarucizumab Aripazin	Andexanet alfa * Aripazin	Andexanet alfa * Aripazin	Andexanet alfa * Aripazin

LMWH, low molecular weight heparin; CYP, cytochrome P450; P-gp, P-glycoprotein (ABCB1); BCRP, breast cancer resistance protein (ABCG2); * Andexanet alfa (PRT064445 or PRT4445) is a modified recombinant FXa protein targeting oral FXa inhibitors.
